# Life and Writings of Vesalius

**Published:** 1861-01

**Authors:** 


### NORTH AMERICAN

### MEDICO-CHIRURGICAL REVIEW.

JANUARY, 1861.

### Analytical and Critical Reviews.

ART. I.—*Etudes sur Vésale, Précédées d'une Notice Historique sur sa Vie et ses Écrits; Ouvrage Publié sous le Patronage des Médecins Belges.* Par AD. BURGGRAEVE, Professeur d'Anatomie à l'Université de Gand, etc. Gand., 1841. 8vo.

*Andreæ Vesalii Opera Omnia Anat. et Chirurg. Cura Herm. Boerhaave et Bern. Sing. Albini. Lugd.* Batav., 1725. 2 vols. fol.

THE author of the first volume at the head of this article properly remarks that there are men who, from the grandeur and wide scope of their genius, do not belong to any particular epoch of the development of the human mind, but seem to comprise it in its entirety, and to constitute, as it were, its eternal symbol. By them every idea bearing the semblance of novelty has been conceived; every discovery has been, if not made, at least foreshadowed. Their history is that of the science to the progress of which they have devoted their powers. Such is the character of the privileged few to whom Providence has intrusted the mission of extending the sphere of the human intelligence. At their approach light assumes the place of darkness; the causes of the phenomena of the natural world and the laws by which these are governed cease to be a mystery. In a word, science is created.

Such were Bacon, Galileo, Descartes, Newton, Lavoisier, Bordeu, Bichat, etc., and such also was the renowned individual who forms the subject of these pages, Andreas Vesalius, to whose genius human anatomy is doubtless more deeply indebted than to any other of its numerous votaries. Anteriorly to him, indeed, human anatomy cannot, strictly speaking, be said to have existed. Among the ancients, contact with or the simple aspect of a dead body imparted an amount of defilement which numerous ablutions and a multitude of other expiatory practices could scarcely efface. On this point the law was in strict harmony with the precepts of religion. We read in holy writ:—

"He that touches the dead body of any man shall be unclean seven days." "He shall purify himself with it on the third day, and on the seventh day he shall be clean; but if he purify not himself the third day, then the seventh day he shall not be clean." "Whosoever toucheth the dead body of any man that is dead, and purifieth not himself, defileth the tabernacle of the Lord; and that soul shall be cut off from Israel: because the water of separation was not sprinkled upon him, he shall be unclean; his uncleanness is yet upon him." "This is the law, when a man dieth in a tent: all that come into the tent, and all that is in the tent, shall be unclean seven days."*

We know with what zeal the Egyptians applied themselves to place the remains of their relatives and friends beyond the reach of decomposition. It is true that the process of embalming, to which they resorted for that purpose, and in which they excelled, required, on the part of the operator, the incision of the large visceral cavities, in order to introduce into these the balsamic substances used on such occasions. But from those operations the science of anatomy was not the gainer, inasmuch as they were intrusted to vulgar and ignorant men, perfectly incompetent to draw useful conclusions from what they saw. Besides, such individuals often performed their duties at the peril of their lives; for, as we learn from Diodorus Siculus, they were apt to be stoned by the populace, as if to make them pay a penalty for the profanation of which they had been guilty.

The same respect to the remains of their dead was paid by the Greeks and Romans. Influenced by the belief that the soul when released from its material envelope was compelled to roam along the shores of the Styx until the body was consigned to the earth or consumed by fire, no consideration could have induced the former to abstain from the performance of that pious duty and permit the dissection of such bodies for scientific or other purposes. It were strange, indeed, if such had not been the case. A belief of the kind and practical anatomy are antagonistic. Hence the Greek physicians and philosophers were compelled to seek such notions of the structure of the body as they desired to obtain, apart from man. Not even Hippocrates sought in the dissection of the human body a knowledge of anatomy; and if he and his countrymen succeeded in acquiring a certain amount of knowledge on the subject, that knowledge was after all only approximative, and was obtained through means of the examination of

* Numbers, xix. 11, 12, 13, 14.

lower animals. That there existed an impossibility on the part of the Greeks to acquire a practical knowledge of anatomy we may infer from the language of Aristotle: "Sunt enim hominum inprimis incertæ atque incognitæ (partes interiores.) Quamobrem ad cæterorum animalium partes, quarum similes sunt humanæ referentes, eas contemplari debemus."*

In the famous school of Alexandria, founded in the third century B.C., under the auspices of the first Ptolemies, anatomy assumed a rank among the sciences. It was publicly taught, and the dissection of the human body ceased to be an offense punishable by law. Pliny informs us that their ruling monarchs themselves studied practical anatomy: "Compartum sit in Ægypto, regibus corpora mortuorum, ad scrutandos morbos, insecantibus."†

The chiefs of this anatomical school, Herophiles and Erasistrates, to whom the science is indebted for the discovery of the lacteal or chyliferous vessels, had a large number of disciples at Smyrna, at Laodicea, and in several cities of Greece; but after their death the science retrograded, their discoveries remained sterile, and once more the dissection of the human body was abandoned. Among the Romans the example set by the school of Alexandria was not followed, and the study of practical anatomy was pursued on the lower animals; and even Galen, whose anatomical writings are the most precise, numerous, and correct of all that have descended from ancient times, very evidently abstained, as was the case also with Rufus of Ephesus, Soranus, Pelops, and others of his contemporaries or predecessors, from dissecting human subjects.

After Galen, no writer added to the store of knowledge already attained on the subject of anatomy. The science indeed retrograded still more, and continued in arrear until the renewal of letters. By the Arabs much was done to promote its advancement, if not in a practical, at least in a theoretical point of view; for although fully aware that anatomy and physiology constitute the basis of every rational system of medical instruction, and although they invariably prefaced the description of every disease by that of the parts principally affected, they were deterred, by the laws of the Koran, from dissecting the human body, and contented themselves with commenting upon the anatomical writings of Galen or with examining the bodies of lower animals.

During the middle ages the dissection of a body made after the image of the Almighty continuing to be regarded in the light of an impious act, subjecting the offender to the most severe punishment, little or nothing was done toward the advancement of anatomical knowledge. The result was

* Hist. Animal, lib. i. cap. 16.

† Hist. Natura., lib. xix.

natural. Knowledge was at that time confined to the monasteries. Even after the establishment of schools—for which the world was indebted to Charlemagne—priests, for a long time, alone possessed sufficient instruction to enable them to consult the writings of the ancients, or impart instruction to those around them. Now, as the Church prohibited in the strictest manner not only all bloody operations, but the dissection of the human body, severe penalties were attached to every infraction of the law. Cassiodorus, who lived in the seventh century, informs us that in his time officers were appointed for the purpose of guarding against the violation of graves. The Salic law, which was the constitution of the Francs and of other German tribes, interdicted the commerce of men to all individuals by whom a body had been exhumed, until the parents had received ample satisfaction.*

This state of things continued, as we have seen, not only up to the ascendency of the Arabian school, but during the period of its greatest influence, from the eleventh to the thirteenth centuries. Nevertheless, the early part of the latter century marks an era of progress; for in 1213 Frederick II., King of the Romans and of the Two Sicilies, prohibited the practice of surgery to those who had not undergone an examination on anatomy; and to facilitate the study of this branch, ordered that in the schools of Sicily and Naples a body should be publicly dissected at least every five years.† Little, however, was done toward the full execution of this law until 1306, more than half a century after the death of Frederick, when Mundinus, Professor of Anatomy in the University of Bologna, publicly dissected the body of a female. Ten years after he dissected two. But here the practice ceased. The scandal was not repeated, and Boniface IV. having launched an edict against those who ventured to violate the dignity of man by dissecting a human body, the example set by Mundinus was not followed, and the science was once more deprived of the only means calculated to insure its progress. It may be remarked that, notwithstanding the facilities which Mundinus possessed of advancing his knowledge in relation to the subject it was his province to teach, the treatise he published on anatomy, popular as it was during two centuries, is little more than a reproduction, often in obscure and misty language, of the anatomy of Galen. To form an idea of the limited extent of the knowledge that could be obtained at that time relative to the structure of the body, we need only refer to the statement of Guy de Cauliac, that the whole course of instruction was embraced in four lessons: the first, on the viscera; the second, on the nervous system; the third, on the muscles; and the fourth, on the limbs.‡

---

* Lex Salica, tit. lvii. art. 5.

† Codex legem antiquarum Lindenbrogi. Francfurti, 1613.

‡ Guy de Cauliac. Anat., cap. i. doctr. 1.

It would appear also that recourse was had to dry preparations, for we are told by Mundinus that to reach the deep muscles of the extremities it is necessary to dry the body in the sun during three years.

During the two following centuries, the fourteenth and fifteenth, the progress of anatomy was very limited, if not null. The dissection of a body was considered as so extraordinary an event, that it was chronicled by contemporary writers as worthy of being transmitted to posterity. Thus we are informed, in a work published in 1546,* that on the 8th of February, 1429, an individual was dissected at Bergamo; and that in 1430 the uterus of a woman who died at Venice was there dissected. Nevertheless some discoveries were made at this period which go to show that the study of anatomy had not been entirely fruitless. Nothing of the kind, it is true, is to be found in the writings of Gabriel Zerli,† Nico Massa,‡ John Gonthier d'Andernach,§ Andrea Laguna,|| etc.; for they contain nothing worthy of notice, and are besides deeply tinctured with the erroneous views of the day. But if from these we turn to Alexander Achillini, Professor of Anatomy in the University of Bologna, matters appear to assume a brighter aspect. This writer, though scarcely less than Zerli wedded to the method and prejudices of Mundinus, and a strong defender of the doctrine of Averrhoës, is entitled to an honorable mention as the discoverer of the olfactory nerves, and of two bones of the internal ear, the malleus and the incus, which he described in a work which appeared in 1516.¶

From the date of the publication of Achillini's work the state of ignorance here referred to began to pass away. The three great discoveries of gunpowder, printing, and the New World, which took place in the space of less than a century, imparted a new tendency to the destiny of the human species The ruling powers of the Church gave their sanction to, nay favored, the pursuit of that branch of anatomy which is indispensable to painters and sculptors. Albert Durer in 1525 published a work illustrating the symmetry of the body—of course as an artist rather than as an anatomist. Under the protection of Julius II. and Leo X., Michael Angelo, Raphael, and Leonardo da Vinci copied from nature the superficial muscles. The taste for the study of anatomy began to spread, while the public at large gradually became familiarized with the idea

* Ars Chirurg. Ven. fol. Apud Juntas. p. 229.

† Anatomia Corporis Humani. fol. Venet, 1502.

‡ Anatomiæ Liber Introductorius. 4to. Venet, 1536.

§ Anatomicarum Institutionum. Liber iv. 8vo. Basil, 1536.

|| Anatomica Methodus. 8vo. Paris, 1535.

¶ Achillini, Corporis Humani Anatomia. 4to. 1516. Annotationes Anatomicæ in Mundinum. fol. Bonon, 1522.

of human dissection. Indeed, the sixteenth century, the most prolific in great and important discoveries, may in like manner be regarded as the one in which the knowledge of the structure of the human body made the most rapid progress. Never, says the learned Sprengel, were there assembled within the same space of time so large a number of illustrious men engaged in efforts to perfect the science of anatomy, the most essential of all to those who devote themselves to the study and practice of medicine.

"The sixteenth century had just opened, and with it the spirit of examination had begun to emancipate human reason, by freeing it from the shackles resulting from the numerous prejudices which heretofore had formed an obstacle to its progress. Among all the sciences, anatomy was one of the very first to enter the course of observation which was opening before it. The ardor displayed in its pursuit was the greater from the fact of the obstacles that had been placed in its way. Anatomical theatres were opened to the public, and could scarcely suffice to accommodate the large number of pupils that flocked therein for the purpose of enjoying a spectacle so new to them. Alexander Benedetti, who at that time taught anatomy in the University of Padua, complains of the inconvenience he experienced from the immense classes that pressed in his large lecture-room.* Another professor equally celebrated, Berengario de Carpi, informs us that from 1502 to 1527, during which he taught anatomy in the Universities of Pavia and Bologna, he dissected more than one hundred bodies.† Nevertheless, no one, up to that time, had ventured to touch the system of Galen. Everything he had taught—errors and truths—was admitted with the same religious respect. In vain was it perceived that his descriptions did not accord with the results of dissections. Convinced of his infallibility, the most hazardous supposed that the text of his works had been corrupted. Others, more fanatic, affirmed that it was nature that had changed, and that the body of man was not then constructed as it was at the time of the great physician of Pergamus. Far from aiding the progress of the science, dissections had the effect of retarding it, raising as they did, at every step, difficulties which it was impossible to remove. Who could dare to set himself up in opposition to a system admitted during the long period of fourteen hundred years? What man will be powerful enough to overthrow dogmas protected by the prescription of ages? That man will be Andreas Vesalius, a youth of twenty-three years, to whom, to use the language of Senac, will be given the privilege of discovering a new world."—*Burggraeve*, pp. 14, 15, 16.

But were we disposed to stop short of this opinion, we cannot refuse to admit with Sprengel, that, if Vesalius does not place himself at the head of all who applied themselves to anatomical pursuits during the period in which he flourished, it cannot be denied that he was the most celebrated, and is entitled, more than any other, to the thanks of posterity, as being

---

* Alex. Bened., Phys. Anat. Bas. 1527.

† Isag. in anat. corp. hum. Bon. 1522.

the first to protest against the blind and slavish submission to which we have referred.

This celebrated anatomist was a native of Brussels, the capital or principal city of the country which, at that time, shared with Italy the honor of being the richest and most enlightened in Europe. The day and year of his birth are not positively ascertained. According to some authorities, he came into the world on the 30th of April, 1513; while, by others, the time is affirmed to have been the 31st of December, 1514. Vesalius, like Hippocrates in ancient times, belonged to a family in which the practice and teaching of medicine was hereditary. Deriving its origin from Wesel, in the Duchy of Cleves, the family, whose name was originally Wittings, assumed that of Wesele or Wessale, which was in time transformed into Vesale or Vesalius. The father of the subject of these pages, whose Christian name was also Andreas, was attached, as apothecary, to the household of the governess of the Lower Country—the Princess Margaret, aunt to the Emperor Charles V. His grandfather, Evvrard Vesalius, acquired a high reputation for his mathematical attainments, and as the author of several medical works, among which may be cited Commentaries on Rhazes, and on the first four sections of the Aphorisms of Hippocrates. The father of Evvrard, John de Wesele, was physician to the Emperor Maximilian, and successively Professor and Rector of the University of Louvain. Finally, the father of John, Peter de Wesele, was in like manner a physician, and enjoyed as such a distinguished reputation.

Andreas Vesalius was early sent to the University of Louvain to pursue his classical studies, and there became well grounded in the Latin, Greek, and Arabic languages, as also in the physical and mathematical sciences. After the completion of these studies, Vesalius proceeded to Montpellier, and subsequently, in 1532, to Paris, for the purpose of applying himself to the acquisition of medical knowledge, and especially of devoting his attention to the study of anatomy and surgery. It must be borne in mind that, at the period mentioned, the facilities for teaching the first of these branches—anatomy—were far inferior to what they are at the present day. It is true, that in the first of these schools, whose celebrity arose principally from the fact of its being the depository of the doctrine of the Arabs, human anatomy constituted an object of serious attention. So early as the year 1376, when dissections were never resorted to in any part of Europe, save Italy, the physicians of Montpellier had obtained from the Governor of Languedoc permission to dissect annually the body of a criminal for purposes of demonstration, which permission was confirmed successively in 1377 by King Charles of Navarre, in 1396 by Charles VI. of France, and finally in 1496 by Charles VIII. Similarly restricted opportunities were offered in Paris. But in neither place could they be of any avail; for though dissections were tolerated, they were either omitted completely or resorted to with the utmost reserve. The prejudices in relation to them had not entirely subsided, and the conscience of the anatomist was not entirely at ease respecting the kind of profanation he regarded himself as compelled to commit when he applied the knife to the human body.

"The course (of anatomy) was limited to commentaries on Galen, to the dissection of some animals, and now and then to that of a human body, performed, in presence of the Professor, by assistants selected for those demonstrations, which were never prolonged beyond three days. Vesalius was not long in attaining a distinguished position among his fellow-students for his bold and enterprising spirit. He was frequently seen in the Cemetery of the Innocents, or on the height of Montfaucon, contending with famished dogs for a prey already in a state of putrefaction. Not less frequently did he return to the lecture-room after the demonstration, and there, taking advantage, for his own sake and that of his friends, of the superiority he had acquired, repeat the lesson, and often correct the errors, of the master. More than once Sylvius (De la Boe, at that time professor of anatomy,) re-entering the room suddenly, found the young auditory occupied in recapitulating the demonstrations under the direction of their fellow-student."—*Burggraeve*, p. 21.

It may not be uninteresting to present here a few remarks relative to the nature of the prelections of Sylvius, and the extent of the information he conveyed to his pupils. Enjoying, as he did at the time and as he continued to do a long time after, a reputation second to few, if any, and superior to most other professors of anatomy, his teachings must naturally be supposed to have been the best, or among the best, that could be obtained. Viewing them, therefore, in that light, some idea will be formed of the manner in which matters must have been conducted in those respects in the various schools of Europe, especially those of an inferior note, up to the middle of the sixteenth century. We use freely the words of a spicy contemporary. Sylvius read to his class Galen on the "Use of Parts." He began fairly, and when he had reached the middle of the first book, at the point where the anatomy commences, he said: "Gentlemen, we now come to a part too difficult for the comprehension of beginners. Were I to go through it with you we should only be bewildering each other." To save trouble, therefore, the Professor took a flying leap over all intervening matter and descended on the fifth book, through which he cantered quietly to the tenth section. From the rest of the work he made selections, to the consideration of which he either gave a single lecture, or to which he devoted five or six lessons at most. This course of professional study was illustrated sometimes with the dissection of some portion of a dog, prepared for the purpose by a surgeon under the Professor's eye. This always was thrown away on the third day, when it became unpleasant to the smell. Sylvius believed, like his brethren, that the anatomy of all flesh was contained in Galen. If he found anything in his dog that puzzled him, the fault lay always with the animal; the dog was wrong. Nay, in his endeavors to explain the discrepancies pointed out to him as existing between the condition of certain parts of the human body and the description of those same parts as given by Galen, he did not hesitate to affirm that the cause was to be sought in the fact that the human species had degenerated since the days of that great writer. Often the learned man—more used to turn over leaves than strips of muscle—blundered about his little preparation, vainly searching for some blood-vessel or tendon that he meant to show. At one of these demonstrations, witnessed by Vesalius, the Professor was so much surprised at the confused construction of the animal before him that he called on his pupil to help him through his difficulty.

Vesalius soon surpassed his teachers, one of whom, already mentioned, Gonthier d'Andernach, who had foreseen the future renown of his pupil, did not hesitate, in 1538, and when the latter had scarcely attained the twenty-fifth year of his age, to confide to him the superintendence of the publication of his works. Hence, from the outset of his career, Vesalius may be said to have proved himself the true offspring of his works. Notwithstanding the high reputation to which his masters had attained, he placed himself by their side rather as their equal than as their pupil. He aided them in their labors, and detected errors which the former, following as they continued to do a blind routine, did not cease to commit in their lectures and writings.

In consequence of the war which broke out between Charles V. and Francis I., Vesalius was obliged to leave Paris, and, returning to Louvain, soon obtained permission to deliver public lectures on anatomy.

Toward the year 1535, Vesalius, who, although little more than twenty years of age, was attached in a professional capacity to the army of Charles V., revisited France, and had there an opportunity of dissecting, for the first time, a human body; for heretofore he had assisted but twice at operations of the kind while prosecuting his studies in Paris. Thence he proceeded to Italy, and in 1537 was invited by the Senate of Venice to the Chair of Anatomy in the University of Padua. Vesalius had long before perceived that the descriptions of Galen did not accord with the results obtained from the dissections of the human body; but distrusting the testimony of his own eyes, he feared lest he might be laboring under the effect of an error of observation. Thus he informs us that he had already commented, three times in his lectures, upon the work of the Greek physician, without daring to point out the numerous errors he had discovered it to contain. Such was the strength of the faith he reposed in the man whom he considered, after Hippocrates, as the greatest and most infallible of physicians! But when his opportunities for dissection were greatly multiplied, when he was enabled to compare the human body with those of lower animals, he could no longer resist the evidence of the facts which presented themselves to his observation; and came to the conclusion that the anatomy of Galen has no relation whatsoever to the human body, but to that of the mammifera which approximate most to man in point of structure—the monkeys. Perceiving this, he lost all confidence in an authority which had so long been his guide, and determined to investigate the entire structure of the human frame and reconstrue the science of anatomy. In view of this, each part was examined with extreme care, and every time he discovered anything which did not accord with the text of Galen, he noted it on the margin of the volume. In this way was composed his great work on anatomy, the production of a youth of twenty-eight, which was designed to revolutionize completely the science and give it the impulse it has continued to this day to obey.

Vesalius resided seven years in Italy. During this period he taught anatomy, not only in Padua, where, as we have seen, he had been invited, but also in Bologna and Pisa. To the latter place he proceeded, at the solicitation of the Grand Duke of Tuscany, Cosmo de Medici, not with a view to add much to his pecuniary resources, the salary being only 600 crowns, but in consequence of the facilities he was there promised in the prosecution of his favorite researches. In this way he was not attached exclusively to either of those establishments, but passed from one to the other to deliver in each a course of lectures, which usually occupied seven weeks.

Of the talents and success of Vesalius as a teacher, Henry Morley, in his delightful biography of Cardano—a work in which he has embodied a mass of interesting materials, collected from a variety of sources—speaks in high terms. Vesalius, he says, was perhaps the only medical teacher in Italy who was then able to fill his lecture-room. He had a stimulating subject. His dissections of real human bodies attracted the curious as much as the inquiring. He was a man of the world, too, strong-willed, and, perhaps, overbearing in his temper, but of courteous habits; young, handsome, well-dressed, affable, and a fluent speaker, master of an admirable style.*

The intervals between these courses of lectures he spent at Venice. There he prosecuted with indefatigable zeal his anatomical studies, at the same time practicing medicine and surgery, and devoting much attention to the composition of his work; which, though commenced during his profes-

* The Life of Girolamo Cardano, of Milan, Physician, vol. ii. pp. 16, 17.

sorship at Padua, did not see the light till the year 1543, in consequence of the extreme care he devoted to the confection of the plates.* Resigning his professorship, he proceeded to Bâle, where his work was being printed, and next returned to his native country, where he had been invited by the emperor. Soon after his arrival he was sent, as surgeon, to the army (1543), and made some stay at Nimeguen, to attend on the Legate of Venice, who was there confined by a severe illness; and thence proceeded to Ratisbon to join the emperor. He there published a dissertation on the virtues and properties of the china (Smilax china) through means of which the emperor had been restored to health. It may be remarked, that this dissertation is rather a work of criticism than one of materia medica; for the observations relative to the plant occupy a smaller space than the defense of the author against the attacks of his antagonists.†

His work on anatomy, which, as already seen, was published this same year, produced a great sensation, and its appearance was the signal of the most violent attacks. This result could not but be anticipated by the author and his friends; for his views and tendencies were well known, as they had been proclaimed in his lectures, and none was ignorant of the fact that they were of a nature to compromise the reputation of many men occupying high positions in the medical hierarchy.

In a small manual of anatomy‡ which he published at this time, and dedicated to Prince Philip, son of Charles V., and which was intended as a kind of prospectus or *table raisonnée* of his great work, he endeavored to prepare the public for the innovations he was about therein to introduce in the science. Finally, in the dedication to the emperor, of the work itself, he explains the necessity for a new treatise on anatomy, and on the right he possessed of preparing it. "Son, grandson, and great-grandson of distinguished physicians, it became me to accomplish that task, at the risk of remaining a stranger to the scientific movement which commenced with your reign, and of rendering myself unworthy of my noble ancestors." He recalls his efforts and laborious researches, and exclaims, "Never would I have become an anatomist had I, while studying medicine in Paris, failed to apply myself to dissections, and contented myself with the clumsy manipulations of ignorant surgeons." After enumerating his labors at Louvain, Padua, Bologna, and Pisa, and alluding to the praises he there received, he remarks :—

---

* De Humani corporis fabrica, libri vii. Bas. 1543.

† Epistola ad Ioachimum Roëlants, etc., rationem modumque propinandi radicis chynæ decocti, quo super invictissimus Carolus V. imperator usus est, pertractans, et præter alia quædam, etc. Ratisb. 1546.

‡ De humani corporis fabrica librorum epitome. fol. Basilæ, 1542.

"There, also, shaking off the shackles of teachers and schools, I have endeavored to demonstrate man as man himself. Indeed, where would have been the use of seeking this knowledge in books? Of all that Herophiles, Erasistrates, Marinus, and many other illustrious authors have written, scarcely a few fragments remain; and as to those who have followed Galen, Oribasius, Theophiles, the Arabs, and our moderns, (admitting that they have left behind them something worthy of being read,) they have all contented themselves with compiling, commenting upon, and most frequently disfiguring him in the most ridiculous manner. Is this the respect due to a great writer? Is it in perpetuating his errors that we can pretend to preserve his memory unsullied? What have I done to justify my being accused of having calumniated him? I have always done him justice; but instead of imitating the generality of our physicians, who cannot discover any errors in his works, although Galen often corrects himself—pointing out in one part mistakes committed in others—instead, I say, of following blindly this deplorable example, I have opposed his opinions, and shown, proofs in hand, that the physician of Pergamus dissected not the human body, but the lower animals—particularly monkeys. In this respect Galen was not guilty, since he was deterred by a prejudice stronger than his good intention or genius. The guilty are those who, having the human organs under their eyes, obstinately copy in a servile manner the errors of their idol."

In reference to the mode of teaching anatomy as pursued by the professors of the times, he exclaims:—

"What shall we say of those teachers who from the rostrum repeat emphatically, like so many parrots, what they have learned in books, without even having themselves made a single observation; or who lecture on preparations so monstrously defective, that their spectators would learn much more from a butcher at the stall? *Ita quoque spectatoribus in illo tumultu pauciora præponuntur quam lanius in macello medicum docere posset.*"

"However," he adds, "I am aware that having scarcely accomplished my twenty-eighth year, I shall be regarded as very bold to attack the physician of Pergamus. I feel that I shall be exposed to the stings of those who have not, like myself, studied anatomy conscientiously; and who, although old and bent by age, preserve at the bottom of their hearts sufficient gall not to pardon a young man for having discovered and demonstrated what they, who represent themselves as masters in the science, have not seen."

Among the opponents whom Vesalius encountered on the occasion, one of the bitterest was his old master, Sylvius De le Boe, who could not brook the idea of being convicted of having all along entertained and taught the most erroneous views; and that, too, through the instrumentality of one whom he considered as a mere boy. Besides, Sylvius felt incensed at perceiving that an individual could be bold enough to impugn the infallibility of Galen. This celebrated individual is described by Cardano, in an account of a dinner they sat to in company with Fernelius, as a merry old man of seventy, quite bald, quite little, and full of jokes. As already seen, he was of the old school—worshiping Galen—teaching anatomy from small fragments of dogs, and omitting from his teachings whatever was at all difficult, even in the authority he worshiped. Sylvius, who was furiously endeavoring to hunt down his old pupil Vesalius, as an impious confuter of the word of Galen, followed him to Madrid with his hate, and sought to bribe a Madrid State physician, Cornelius Barstrop, with the promise of a baby's skeleton, if he would join the chase. Persecution of Vesalius had become the topmost thought of his old age, and he could not of course dine with a strange doctor without mounting on his hobby. "He was breathing animosity against Vesalius, says Cardano, arising I know not from what cause. He professed, indeed, that it was for wrongs done to Galen; and he demanded a most iniquitous thing, that I, too, should become his enemy."* In the excess of his wrath, Sylvius published a pamphlet, entitled *Sylvius Væsani Calumnius deplusandus*, in which, taking in hand the defense of Galen, he brands his old pupil with the epithets of calumniator, impious turn-coat, points him out as a monster whose impure breath was poisoning Europe, and descends so low as to launch against him a miserable play of words, which, as some writers properly remark, is fortunately untranslatable. "Vesalius non esse, sed Vesanum." A much more dangerous adversary was found in Eustachius, Professor of Anatomy in the University of Rome, who, like Sylvius, constituted himself the defender of Galen; but who, instead of combating, like the other, his antagonist by heaping abuses upon him or turning him into ridicule, endeavored to overthrow his views by an appeal to facts and arguments.†

Attacks of the sort, proceeding from a man universally and deservedly recognized as one of the first anatomists of the age, naturally called for an answer. For this purpose Vesalius once more visited Italy, in order to demonstrate publicly the erroneousness of the charges brought against him. The University of Padua, where he first proceeded, placed at his disposal all the dead bodies required for his demonstrations. He then summoned his opponents, inviting them by a public notice to that effect, to make their own dissections from his subject, and state their objections, and expressing his readiness to answer these. Need it be added that his triumph was complete—not a man venturing to accept the challenge—and that henceforward no one could deny the correctness of the facts he exposed to light? From Padua Vesalius went to Pisa and Bologna, in each of which places he obtained an equal success.

But while such was the result of his visit to Italy, the clamors and

* Morley, op. cit., vol. ii. pp. 100, 101.
† Eust. Tract. de Ven. Azyg.

every-day accusations of Sylvius and other worshipers of Galen spread over Europe, and revived the prejudices of the public against the dissection of the human body. The excitement produced in the matter became indeed so great that the emperor thought himself under the necessity of ordering an investigation into the contents and tendencies of the incriminated work, and of insuring, if necessary, its censure by the proper authorities. The theologians of Salamanca were summoned to decide the question whether Catholics were permitted to open dead human bodies? Fortunately for Vesalius and for science, the answer of the Spanish monks was in the affirmative.

On being apprised of the order issued by the emperor, Vesalius determined to quit Italy; and immediately, as we learn from Haller, consigned to the flames, in the excess of his mortification, his books and manuscripts. In this way, we are told, were lost a huge volume of annotations upon Galen, a whole book of medical formulæ, many original notes upon drugs, the copy of Galen from which he lectured, covered with marginal notes of new observations that had occurred to him while demonstrating, the paraphrase of the books of Rhazes, presently to be mentioned, etc. That Vesalius soon repented of his deed, we have every reason to believe. But it was too late; the sacrifice was consummated, and with a bleeding heart he retired to Brussels, where he lived for some time in the utmost seclusion. In 1546 he proceeded to Bâle, and there remained long enough to superintend the reprinting of his work, occupying a few hours each day in giving demonstrations in anatomy. In return for the kind reception he there met with, he presented to the medical school of Bâle a skeleton, which is still (or was until lately) preserved therein with religious care, and over which was placed a complimentary inscription. On the abdication of Charles V., Vesalius followed Philip II. to Spain in the capacity of court physician.

From this period may be dated the close of the scientific career of the great anatomist. Thrown in the midst of a court, gloomy and swayed by prejudices, he was far, notwithstanding the respectful consideration with which he was treated, from leading a happy and tranquil life. According to some historians, he became the subject of annoyances on the part of the physicians by whom he was surrounded, who viewed with feelings of jealousy the professional eminence which he, a stranger among them, was enjoying; and did all in their power to drive him away or tarnish his fame. Boerhaave and Albinus also inform us that Vesalius was equally annoyed by the Spanish monks, who made him pay dear for the jests be continually leveled against them on the score of their ignorance, their costume, and their morals. Under the impression of these influences, he became discouraged, depressed, and indifferent to passing events. Another circumstance tended to increase this painful mental condition. Since his arrival in Spain, the impulse he had imparted to anatomy in Italy had considerably increased. In 1562, Francis Puteus, of Vercelli, ranging himself, in a work addressed to him,* among the warmest admirers of Galen, attempted to defend the latter, and to show that he was wrongfully accused of not having dissected human subjects. About the same time the distinguished pupil of Vesalius, Fallopius, who, all things considered, may be regarded as the greatest anatomist of the sixteenth century, published his *Anatomical Observations*, a kind of repertory or critical examination of all that had been previously written on the subject of anatomy, and in which the author, while recording his own discoveries, points out, though in the most respectful manner, the inaccuracies of his illustrious master. Against the statements, criticisms, and aspersions of Puteus, Vesalius defended himself in a pamphlet which was published in 1565, under the assumed name of *Gab. Cuneus*,† and the authorship of which is ascribed to him by his friend and contemporary Cardano.

The above-mentioned work of Fallopius recalled to the mind of Vesalius thoughts of an agreeable, and, at the same time, painful character. It made him revert, as he says in an examination of the work, which he addressed to Fallopius himself, to a period full of glory, when he recognized no master but science, no ambition but its progress—to that period when the whole of Italy applauded his success. He exhibits a contrast between that period and the present, when he no longer possessed the means of keeping himself on a level with the state of the science he had created, and besides experienced the mortification of seeing himself surpassed by his own pupils, without the possibility of his being near by to direct their labors and to rejoice in their progress. He does not conceal his desire of being able to resume his favorite studies, and, in conclusion, remarks:—

> "I hope, should I have, some day, the opportunity of performing dissections—an opportunity which is completely denied me here, where I have not been able to obtain a cranium—I hope, I say, to go over the structure of the entire body, and to review my entire work."

In the composition of this work, which appeared in 1564,‡ Vesalius fell, in some respects, far short of the high reputation he had attained, as is fully admitted by his two illustrious editors, Boerhaave and Albinus: "Aulicis obnoxius, toteis obsequiis, hæret cerebro, vera negat, sœpè minus

---

* Putei apologia pro Galeno. 8vo. Venet, 1562.

† Gab. Cunei Midiolanensis apologiæ Fransc. Putei pro Galeno anatome examen.

‡ Anatomicarum Gabrielis Fallopii Observationum Examen. Venet, 1564. 4to.

proba asserit," etc. Nevertheless, he very naturally attached a considerable importance to the publication of what he viewed as a satisfactory vindication of his anatomy, and intrusted the manuscript to the Ambassador of Venice, Paul Tiepolo, with the request that he would place it in the hands of Fallopius, at Padua. But being retained in Spain by the events of the war, Tiepolo did not reach Venice till the next year, when, being informed of the death of Fallopius, he kept and made no use of the manuscript, which was soon after obtained by the author while at Venice on his way to Jerusalem.

This incident increased the mental dejection of Vesalius, and excited in him the desire of returning to Italy—that native focus of genius, as he calls it, *engeniorum vera altrix*—with a view to defend his reputation, which he thought compromised by the writings of Fallopius. But to leave Spain was matter of no little difficulty, inasmuch as the authorization of Philip II. was required; and the latter, who appreciated fully the importance of the professional services of the great surgeon, was not likely to consent readily to his transferring his residence elsewhere. The only means of effecting the object in question, which offered a prospect of success, was to pretext a desire of fulfilling a religious vow of a pilgrimage to the Holy Land. To this effect permission was sought and finally obtained, and as soon as the preparations for the long journey were made, Vesalius quitted Madrid and proceeded to Venice, where, in company with J. Malatesta de Remini, General of the Republic, he embarked for Cyprus, and thence for Jerusalem.

While there, he received from the Senate of Venice the offer of the chair of anatomy in the school of Padua, left vacant by the death of Fallopius. This offer he gladly accepted, rejoicing in the thought of returning to Italy, and in the prospect of being, at the age of only fifty-eight, able to devote himself once more to scientific pursuits. Quitting Jerusalem, Vesalius embarked for Venice; but the vessel in which he had taken passage was wrecked, on the 2d of October, 1564, on the coast of the Island of Zanthe, where he soon after died. A jeweler, by whom he was recognized, caused him to be buried in a chapel dedicated to the Virgin, and placed over his tomb the following inscription:—

ANDREÆ VESALII BRUXELLENSIS TUMULUS,
QUI OBIIT IDIBUS OCTOBRIS,
ANNO 1564,
ÆTATIS VERO SUÆ QUINQUAGESIMO,
QUUM HIEROSOLIMIS REDIISSET.

Thus, says M. Burggraeve, perished, a victim of his love of science, the extraordinary man who created a science the most vast of all, at a period when every sort of obstacle was thrown in the way of its progress—the man whose entire life was nothing but a long struggle between knowledge and ignorance, between truth and error. He had quitted Spain, not for the purpose of escaping the consequences of a disgraceful condemnation, but to resume the active and laborious life incident to scientific pursuits and professional duties. God only knows how far this ardent genius would have pushed anatomy, seeing that, at the very outset of his career, unaided and amid the most adverse circumstances, he brought it nearly to a state of perfection.

By biographers and historians a number of statements having reference to the position occupied by Vesalius in Spain, to the causes of the difficulties he there encountered, and to the circumstances which led to his pilgrimage to the Holy Land, have been recorded. Several of them, and the last more particularly, deserve to be examined, in order to ascertain how far they are entitled to belief. Thus it is stated that at the close of the year 1543, or the beginning of the following, Vesalius was called to the Court of Charles V., in the capacity of his protomedico or first physician; that he followed the emperor as such to Madrid; and that when this sovereign, weary of the responsibilities of the empire, abdicated, his son and successor to the throne of Spain, Philip II., retained Vesalius in the same position near his person, and honored him with his entire confidence.

It is also mentioned that the Infant Don Carlos, son of Philip II., was attended and cured of an attack of cerebral compression, the result of a fall on the head, by Vesalius, who, in opposition to the decided opinion of the consulting physicians, resorted to the trephine; that his success on this occasion secured him still more firmly the entire confidence of the king, and that this, exciting the jealousy of the Spanish physicians, gave rise to the persecutions he subsequently experienced at their hands.

Again it is stated that Vesalius had the misfortune to open the body of a gentleman whom he erroneously supposed to be dead; that the nature of the disease under which the individual had labored being of an obscure character, permission was sought and obtained to make the examination; that on touching the heart with the end of his scalpel, this organ, which still preserved a marked degree of vitality, contracted; that he was prosecuted by the relations of the deceased, on a charge of homicide and impiety, before the ordinary courts and the tribunal of the Inquisition, by which he was condemned to death, and that this condemnation was, by Philip II., commuted to an expiatory pilgrimage to the Holy Land.

On each of these points we shall make a few comments.

In regard to the first, it may be remarked that the fact to which it refers—the appointment of Vesalius to the post of protomedico to the

Emperor Charles V., and to his successor to the Spanish throne—is far from being well authenticated. That, in 1543, he obtained an honorable post at the court of the emperor and in the army; and that after the abdication of this sovereign, he followed Philip to Spain, are circumstances which have just been noted. But nothing shows that the post he thus held amounted to that of protomedico. Had Vesalius been the personal ordinary medical attendant of the emperor, he would scarcely have been sent in the year of his appointment to the army in the capacity of surgeon, and allowed to remain some time, at Nimeguen, in attendance on the Venetian Legate. Nor would he have gone to Italy to combat Eustachius and his other opponents. Again, he would not, had he held the appointment in question, have been subjected to the mortification of seeing his work referred to the theologians of Salamanca; nor, in the supposition of such a reference having really been made, would he have continued to hold the appointment. Further than this: it is not probable that in the event of his being protomedico of the emperor, he would, on hearing of the reference, have removed to and lived in retirement in Brussels, and have resided a sufficient time in 1546 at Bâle to superintend the republication of his work.

Independently of this, it must be remembered that Vesalius does not assume the title of protomedico of the emperor, or, subsequently, of the king, on the title-page of any of his works, which he would certainly have done had he occupied the post. Had he really been the head medical attendant on Charles, and enjoyed the confidence of the sovereign, he would, in all probability, have followed him to the Monastery of St. Yust in the place of Henrico Matisio, who remained there during the two years of the sojourn of the emperor, and attended him in his last illness. At any rate, he would have been called in consultation, as was the case with D. Cornelio, physician of the Queen Donna Maria, who resided in Valladolid. Again, he would, in all probability, have been among the earliest of the medical attendants, if not the very first, called in to attend the king's son under circumstances presently to be mentioned; whereas, as we shall see, such was not the case. Finally, the signature of Vesalius, as *President*, would be found at the bottom of the ordinances of the Council of Health.

The second statement of which mention has been made—that relative to the agency of Vesalius in the cure of Don Carlos, the operation to which he resorted to attain that object, and the effect his success occasioned—rests on doubtful grounds. Of the accident which befell the prince mention is made, in more or less details, by all the historians and many other writers of the times. It is recorded in the dispatches of the French Ambassadors, Guibert and St. Sulpice, and is described in the accounts of the case drawn up by Daza and Olivares, the surgical and medical attendants on the occasion. Guibert writes, April 3, 1562:—

"'The fever has left the prince, and his health improves, although, in consequence of his foolish doings, symptoms of his former malady remain behind. This will all be removed as the summer advances.' This hope was, however, completely frustrated, when Carlos had his heavy fall down stairs at Alcala. The same writer says, May 15, 1562: 'The lamentation, perplexity, and despair of the king and his suite is incredible. Don Carlos fell head foremost down a dark stair, which he would descend alone and secretly, in search of a young daughter of a porter in the garden, whom he admired. The surgeons, not satisfied with bandaging the wound, have enlarged it.' The new French Ambassador, St. Sulpice, writes, May tenth, from Burgos: 'They mean to trepan the prince, and have ordered solemn processions and prayers for his recovery.'"*

Mr. Prescott, who follows the statement of Dr. Olivares, one of the physicians of the Prince, says:—

"One evening in April, 1562, as he was descending a flight of stairs, he made a misstep, and fell headlong, down five or six stairs, against a door at the bottom of the passage. He was taken up senseless and removed to his chamber, where his physicians were instantly summoned, and the necessary remedies applied. At first it seemed only a simple contusion on the head, and the applications of the doctors had the desired effect. But soon the symptoms became more alarming: fever set in; he was attacked by erysipelas; his head swelled to an enormous size; he became totally blind, and this was followed by delirium. It now appeared that the skull was fractured. The royal physicians were called in, and, after a stormy consultation, in which the doctors differed, as usual, as to the remedies to be applied, it was determined to trepan the patient. The operation was carefully performed; a part of the bone of the skull was removed; but relief was not obtained."†

Another still more different account of the case was published fifty-eight years after the occurrence, by Melchior Adams. We give it entire.‡

"Philip II. of Spain had a son, Charles, (Don Carlos,) grandson of the Emperor Charles V. This youth had been ordered change of air, upon an attack of intermittent fever. During this retreat, he one day espied a young lady of rank sitting in a certain noble residence, employed in weaving flowers into a wreath. In mere sport, the young prince ran toward her; the noble maiden flew to her chamber, and drew the bolt behind her. The youth thereupon being only the more excited in his sport, broke open with main force the door, and fell headlong down the stairway which was immediately behind it, receiving in

---

* Raumer, Hist. of the Sixteenth and Seventeenth Centuries, vol. i. p. 127. 8vo. From Bib. Roy. MSS., Nos. 9746, 9748.

† Relacion de la enformedad del Principe por al Doctor Olivares. Documentos Inéditos, tome xv. p. 554. Prescott's Hist. of Philip II., vol. ii. p. 517.

‡ Vitæ Germanorum Medicorum, p. 132. Heidelb., 1620.

his fall a very severe wound upon the head. Surgeons were immediately called in. They bound up the wound; but being intent only upon healing the wound, they took no pains to allow the extravasated blood to escape. The consequence was that the whole head became uncommonly swollen, and the prince was lying like one that had been struck with apoplexy.

"Then, at length, the king having been summoned to see his son for the last time, (as being about to die,) brought with him Vesalius, whom the Spaniards had been wont to calumniate as one who, inasmuch as he busied himself only with the anatomy of the body, was unworthy to be called a real physician. He immediately gave it as his opinion, that in so desperate a case the only hope of cure lay in making an incision in the scalp. The king having given his consent, Vesalius opened the swelling by a crucial incision. A discharge of fetid matter began slowly to escape; the youth thereupon came to himself as if awaking from a deep sleep, and declared that he owed his restoration to life to the German physician."

Of all that precedes, the point most interesting to us on the present occasion is the important part ascribed to Vesalius in the treatment of the prince, and the resort said to have been made by him to the trephine, contrary to the decided opinion of the other professional attendants. Considering that had Vesalius treated the prince from the outset of the accident, had he retarded so long a resort to the operation, and had he pursued, under the circumstances mentioned in the above account, the course attributed to him, he would have been guilty of bad surgery, it is a source of gratification to find that the charge is far from resting on a sure foundation. Indeed, Hernandez Morejon, whose authority, from the access he enjoyed to the necessary documents, is entitled to the highest consideration, has no hesitation in denying, in his history of Spanish Medicine, the truth of the story.*

The following is the true account of the case according to this writer :—

The prince, as already seen, fell at Alcala, not from a horse, as was stated in an old MS., but in running down an obscure and dilapidated staircase, and, striking his head against a closed door, was picked up senseless. The first dressings were applied by Daza. Subsequently, the prince was treated by Portuguez, who, like Daza, enjoyed a high reputation for his surgical abilities, and nine professors, physicians, and surgeons, were in succession called in consultation. The first day, Daza, Vega, and Olivares saw the patient; the second, Gutierrez de Santander, Portuguez, and Pedro Torres attended. After the removal of the dressings, Mina and Vesalius were called in, and finally, twenty days after the accident, the services of the Bachelor Gorres were required.

Vesalius did not see the patient till the eleventh day after the accident.

---

* Historia Filosofico—Critico de la Medicina espagnola, vol. iii. Madrid.

He expressed the opinion that his highness should be trephined, on the ground that the disease was internal. This advice *was not followed;* and none of the operations which were required during the three months that the disease lasted, (cupping, bleeding, dressings,) were performed by Vesalius. If this illustrious, rare, and profoundly learned man (Este doctissimo, insegne, y raro varon, as his colleague Daza denominates him,) had treated the prince, would no one have been found to write the history of a cure which was calculated to do him so much honor?

The prince himself directed Daza to draw up the account of the case. He was the first summoned, and to him, had it been necessary to use the trephine, Vesalius would have yielded the instrument; for although as an anatomist the latter had great dexterity in dissecting, he was slow and irresolute when he attempted to operate on the living body; and during the time he served as a surgeon in the armies of Charles V., whether in Germany or elsewhere, he almost always intrusted another surgeon, Castillan, with this duty.

Morejon adds, that so far from enhancing the reputation of Vesalius, the disease of the prince was more calculated to mortify him and wound his feelings; not only because his advice was not followed, or his prognosis verified, but because he suffered, in conjunction with his colleagues, from the effect of a tendency frequently encountered by physicians, not only among the vulgar, but also in the palaces of the rich or of kings. Through the interference of some influential persons the plaster of a Moorish empiric, from the kingdom of Valencia, who at that time happened to be at Saragossa, was resorted to. This individual, named Pintaret, was ordered to Alcala on the twenty-first day after the accident, and allowed to apply, himself, his ointment; and if, fortunately for the Spanish surgeons, the condition of the young prince had not become aggravated after the application, they would have been discharged, and Vesalius, together with his colleagues, would, in all probability, have witnessed the installation at court of the empiric Pintaret.

We cannot close the subject without alluding to the fact that, in the account of the case given by Mr. Prescott from Olivares, mention is made of a morbid state not referred to by Morejon, who, as we have seen, follows Daza—we refer to erysipelas. And yet Daza, being the attending surgeon on the occasion, could not have helped, had that disease occurred, to notice it, and it was too important a fact not to have been mentioned in a narrative of the case. Again, while it follows from the statement of Morejon, always on the authority of Daza, that the patient was not trephined, Olivares, as reported by Mr. Prescott, says he was. But whether such was the case or not, Vesalius does not appear to have been the operator; for Olivares does not mention him—a neglect of which he would scarcely have been guilty, considering the celebrity of that individual. We cannot, indeed, believe that Vesalius would have rendered himself amenable to the charge of such bad surgery, as to trephine a patient laboring under a condition of the scalp such as the one described.

Where Melchior Adams obtained all the information which more than half a century after the accident enabled him to write the above account, he does not say. Therein we find that Vesalius was the operator, but instead of being told that he trephined the patient, we only hear of his incising the scalp and letting out a quantity of fetid pus. Would any quantity of such matter, accumulated in or under the scalp, produce an apoplectic state? and could a person in such a condition experience, from an incision by which the matter was allowed to escape, the sudden relief attributed to the operation?

In regard to the third statement circulated relative to Vesalius—the autopsy by him of an individual still alive, his condemnation to death by the civil tribunals and by the Inquisition, on the charge of homicide and impiety, the commutation of the penalty to an expiatory pilgrimage to the Holy Land, and the fact that the prosecution was instituted at the instigation of Spanish physicians envious of the high position he enjoyed, much has been said. Dr. Burggraeve properly remarks, that before admitting statements calculated, if true, to injure the reputation of Vesalius, or to throw, if false, odium on the character of the Spanish physicians, it becomes a duty to examine how far they are founded, and whether the facts on which they rest are entitled to respectful consideration. Now if acting in accordance with this rule we investigate the statement in question with the care it so richly deserves from the high professional position of the accused, and the gravity of the charge brought against him, we shall not be long in arriving at a conclusion somewhat different from that adopted by the generality of medical readers.

We must premise that the story appears to have originated with, or at any rate cannot be traced beyond Hubert Languet, a controversial political writer of the time, of whom De Thou speaks in high terms, and who was, at the date of his writing, not in Spain—the scene of the supposed event, and where, indeed, he never went—but in Paris.

The information was conveyed by that writer, in a letter dated January, 1565, to his correspondent Gaspard Peucer, the learned physician and mathematician, and thence, after having been circulated freely, found its way in Melchior Adams's "History of the Lives of German Physicians," already mentioned.* "It is said," to use the words of Languet, "that Vesalius is dead. You have doubtless heard that he had undertaken a

* Vitæ Germanorum Medicorum. Heidelberg, 1620, p. 133.

journey to Jerusalem. I learn from Spain the cause of his journey, which is really surprising," etc., (then follows the story of the dissection, etc.)* On the testimony of this communication, the statement was related by De Thou† and various other writers, and, what was of greater importance as regards its fate, adopted by Boerhaave and Albinus, in their life of Vesalius prefixed to their beautiful edition of his collected works, and through them accredited, from that time to this, by scientific men, biographers, historians, reviewers, and others, who, referring to the above authorities, or more generally copying each other, represent the occurrence as one placed beyond the possibility of doubt.‡

The greater number of these writers adopt the story in all its details. Others, as for example, Richerand and Pettigrew,§ while doubting the truth of the individual subjected to the autopsy being alive at the time, seem to admit the reality of the story so far as regards the operation and the subsequent condemnation; and endeavor to explain how the belief in the continuation of life, on which the condemnation was based, could have occurred. We may suppose, according to the first of these writers, that one of the assistants, by leaning on the body, caused the blood to flow to the auricles, whence resulted an obscure tremulousness or undulatory movement, and that this mechanical effect being mistaken for a sign of life, caused a cry of alarm among the spectators, which was repeated by the

---

* Fama est, Vesalium esse mortuum. Audivistis procul dubio, eumprofectum esse Hierosolymam. Causa istius peregrinationis est mirabilis; ut ad nos ex Hispania est perscriptum. Commissus fuit curæ ejus quidam vir nobilis in Hispania; quem eùm obiisse existimaret; nec satis percepisse causam morbi sibi videretur; petiit à propinquis defuncti, ut sibi liceret cadaver dissecare. Concessum est ei, quod petebat; cumque pectus opertuisset: reperit cor adhuc palpitans. Cognati mortui non contenti eum accusare factæ cædis; accusant ætiam impietatis apud Inquisitionem; existimantes, se ibi severiorem ultionem consecuturos. Cùm constaret de cæde; nec facile excusaretur error tamperiti medici: voluit omnino Inquisitio de eo sumere supplicium: vixque potuit rex suâ autoritate, vel potiùs suis precibus eum periculo tanto eripere. Tandem concessus est regi et toti aulæ, pro eo deprecanti, eâ conditione: ut ad expiandum illud scelus proficisceretur Hierosolymam: et ad Montem Sinai.

Datæ Latetia Jan., 1565. Hæc Languetus.

† See the above work; see also Iac. Aug. Thuane Historiarum Sui Temporis, vol. ii. lib. 36, p. 398, French trans.; vol. iii. p. 492, note.

‡ Melchoir Adams, op. cit. Moréri, Le Grand Dict. Hist., x. p. 560. Biographie Médicale. vii. p. 420. Bayle et Thillaye, Biogr. Médicale par ordre Chronologique, i. p. 231. Encyclopédie Méthodique, xiii. p. 429. Encycl. Britan., xxi. p. 611, 7th ed. S. H. Dickson's Essays on Life, Sleep, Pain, and Death. Phila. 1852. Frazer's Mag , Nov. 1853. p. 500. Professional Anecdotes, iii. p. 225.

§ Michaud, Biographie Universelle, xlviii. p. 308, Art. Vésale; Medical Portrait Gallery, ii.

enemies of Vesalius who were but too happy to seize so good an opportunity to do him injury. On the other hand, Pettigrew refers to the possibility that the muscular fibres of the heart acting by their principle of irritability—a principle unknown in the time of Vesalius—remaining even after vitality had quitted the body, may have tended to sanction the statement made.

Another set of writers who have investigated the question with care, or who were placed in a position enabling them to arrive at just conclusions on the subject, have either doubted the truth of the statement, or denied it from beginning to end.

We have no hesitation in ranging ourselves under the banners of the latter set, and in avowing the belief that the whole story—dissection, trial, condemnation, and all—is destitute of foundation. In the first place, it must be remarked that Languet, who, as we have seen, resided hundreds of miles from the scene of the pretended occurrence, reports it not as one of the truth of which he was certain, but as being communicated to him by some correspondent he does not name, and for whose veracity he does not vouch. It has, therefore, the appearance of a mere *on dit;* and we cannot but think it were a hard case if the professional reputation of a man so eminent as Vesalius could be blasted, and be handed down to posterity obscured by a stigma of the kind, on the strength of a report picked up about the streets of Madrid by a gossiping correspondent, and communicated in a letter to a distant friend. Sprengel, while speaking of Vesalius in his great history of medicine, so far from expressing a belief in the truth of the story, remarks that the reason which induced the great anatomist to undertake a visit to Jerusalem is diversely stated; and adds, that at the present day it is impossible to decide which of those opinions approximates most to the truth.* Lampillas, whose testimony, from the circumstance of his being unconnected with the medical profession, and besides that a Spaniard, cannot, in a case of this kind, be impugned, says nothing of the pretended autopsy, trial, and condemnation, and, like many others, attributes the departure of Vesalius for Jerusalem to a desire, on his part, of fulfilling a vow.† Dutith de Horakoweiz, as quoted by Sprengel, (vol. iv. p. 6, note,) expresses a similar opinion.‡ De Thou says much the same thing.

Morejon, in his history of medicine in Spain, goes much further, and denies the story in most positive terms, on grounds which we think are entitled to the utmost consideration.

---

* Histoire de la Médecine, iv. pp. 5, 6.

† Saggio Storico Apologetico della littérature Spagneola, etc., ii. p. 247.

‡ Craton. Epist., lib. iii. p. 212. § Op. cit., iii. p. 492.

"Those," he says, "who invented it, did not think of proving it. What was the name of the individual whom Vesalius dissected before life was extinct? Who were the witnesses that proved the fact before the Inquisition? By which of the tribunals then existing in Spain was the case tried? How does it happen that Don Antonio Lorente, who, as already seen, refers to Vesalius, makes no mention of the trial in question in his annals or critical history of the Inquisition? How comes it that writers of the time of the death of Vesalius, and some, indeed, who were his colleagues at the Spanish Court, are silent respecting an event which, if true, would doubtless have fixed their attention, and of which they would have spoken; some to pity him, others to cast blame upon him, and all to entreat the clemency of the tribunal or of the monarch? Why? Because the fact is absolutely false."

To this Morejon adds, that, so far from Vesalius having been the subject of envy on the part of Spanish physicians and by them persecuted, the professors of that country sang his praise in the loudest tones, and chronicled his profound learning and his dexterity in anatomy. The unanswerable proofs of this are to be found in the writings of Valverde, of Pedro Ximeno, of Collado, of Daza, and of a large number of others, among whom may be cited Alphonzo Rodriguez de Goevara, Professor of Anatomy at Valladolid, and subsequently physician of the Queen of Portugal, Dona Catherina, who, while defending Galen, praises the anatomical knowledge of Vesalius.

It is a circumstance worthy of being noted, that the accounts given of this pretended event differ so materially from each other as to destroy the reliability to which they might otherwise be entitled. According to one version, Vesalius attended professionally a Spaniard of high rank for a disease, the nature and seat of which he had not been able to ascertain. At the death of the patient, he sought and obtained permission to make the autopsy of the body; but as soon as the heart was exposed it was found to beat and palpitate. Ambrose Paré, writing in 1562, that is, at the very time when the event is supposed to have occurred, has a different story on the subject, as may be seen in his chapter on the *suffocation of the womb*. After describing this disease, and the apparent death to which it may give rise, Paré adds:—

"Partant en cette disposition ne faut se haster les ensevelir, et moins ouvrir leur corps, de peur d'encourir une calomnie, ainsi que de ce siècle est arrivé à un grand anatomiste. Ie dis grand et célèbre, de qui les livres réparent aujourd'hui les études des hommes doctes; lequel étant pour lors résidant en Espagne, fut mandé pour ouvrir une femme de maison qu'on estimait être morte d'une suffocation de matrice. Le deuxième coup de razoir qu'il lui donna, commença la dite femme à se mouvoir et à démontrer par d'autres signes qu'elle vivait encore. Je laisse à penser au lecteur comme ce bon seigneur faisant cette œuvre, fut en perplexité et comme on cria *Tolle* après lui. Tellement que tout ce qu'il put faire, fut de s'absentor du pays, car ceux qui le devaient excuser étaient ceux couraient sus. Et estant exilé, tost après mourut de déplaisir; c'est ce-qui n'a pas été sans une grande perte pour la république."*

In noticing the discrepancy presented by these two versions of the same story, Burggraeve inquires how far it is probable that a man so profoundly learned as Vesalius would have ventured to open a human body so soon after the cessation of the phenomena of existence as to justify the presumption that life was not yet extinct. How, especially, can we admit the truth of the first version, when, as Richerand well observes, we consider that before the heart could have been exposed or even reached, it was necessary to have recourse to long and deep incisions, well calculated to recall life if still existing? In the second case, the supposed victim must have labored under an attack of epileptiform hysteria, with loss of consciousness, for the suffocation of the womb of Paré was no more than this. But Vesalius was too well aware of the precautions necessary to be taken, under circumstances of the kind, to open the body of a person laboring under the disease, during the continuance of the paroxysm.

Add to this that Ch. Delécluse, (Clusius,) the celebrated botanist, having arrived at Madrid at the very time when Vesalius left that city on his way to the Holy Land, must assuredly have been able to learn the true cause of his departure. And yet, on receiving the volume of the history of De Thou, in which mention is made of the death of Vesalius, he hastened to write to this historian for the purpose of pointing out some errors which the writer had committed relative to the cause of that event. His communication, which appears to have induced De Thou to modify his opinion on the subject, was inserted as a note in the subsequent editions. He informs De Thou that, remaining in Spain against his will, Vesalius fell into a state of melancholy, and finally became seriously ill of a disease from which he recovered with great difficulty. In consequence, he pressed the king for permission to leave Spain, in order to be able to fulfill a vow he had made during his illness, to undertake a pilgrimage to the Holy Land. "I have learned these details," says Delécluse, "from Ch. Tisenau, chief of the council of the Low Countries, at Madrid."†

But enough, we think, has been said on this subject to justify the opinion we have expressed.

It remains only to remark that besides the works already mentioned, Vesalius was the author of several of more or less importance and of greater or less size. Indeed, when we consider the amount of his labors as a writer, and bear in mind the circumstances under which most of that

---

* Œuvres de Ambroise Paré, liv. xxiv. chap. liv. p. 627; Paris, 1685. Malgaigne, ed. 2, p. 755.

† De Thou, Histoire Universelle, vol. iii. p. 491, 2d note.

labor was performed, and the short space of time—less than thirty years—during which the whole was effected, we can have no hesitation in admitting that few men have exhibited so great a degree of mental activity. Without entering into details relative to the collation of the Greek text of Galen, which he undertook for the great Venetian printer Junta, or to the paraphrase of the work of Rhazes, already mentioned, in which the knowledge of the Arabians is compared with that of the Greeks and other writers,* and which was prepared for his inaugural dissertation, we may state that Vesalius published a new edition of the "Institutions" of his old master, Gonthier d'Andernach, which he revised and corrected with the greatest care, and at the cost of much time and labor. In 1539 appeared a letter on the necessity of bleeding in the treatment of pleurisy.† The next year he issued at Venice his first anatomical plates, engraved on wood. They were intended as a kind of specimen of the fine plates which were to illustrate his large work on anatomy. From 1540 to 1564 Vesalius composed a large number of written consultations on a variety of medical and surgical diseases, which may be found in the works of Ingrassias, Montanus Scholtzius, etc.

His great work on surgery, though completed as early as 1561, did not appear till some time after his death. We are informed that by a concourse of fortuitous circumstances the manuscript of this work found its way, after this event, into the hands of a bookseller in Paris. By him it was sold to a physician named Bergarutius, who edited it and published it at his expense.‡ M. Burggraeve remarks that this physician has been taxed with having been influenced in this transaction by motives of pecuniary interest. By some, indeed, the work has been regarded as a mere fraudulent operation. But there is no more reason to doubt its authenticity, than that of the other writings of the same author.

A collected edition of the works of Vesalius was published in 1725 by Boerhaave and Albinus, with an introduction and life of the author by the celebrated editors.§

Thanks to the impulse imparted by Vesalius to the investigation of anatomical subjects and the death-blow he had given to the authority of Galen so far as regards this branch of knowledge, there sprang up about the time of or not long after his death a whole generation of anatomists,

* Paraphrasis in nonum librum Rhazæ ad Almamsorem de affectum singularium corporis partium curacione. Bazil, 1537.

† Epistola docens Venam anxilarem dextri cubiti in dolore laterali secundam, etc. Basilæ, 1539. 4to.

‡ Andreæ Vesalii Chirurgia Magna in qua, nihil desiderari protest, quod ad perfectum, etc. 8vo. 1560.

§ Andreæ Vesalii Opera Omnia Anat. et Chirurg. Cura Herm. Boerhaave et Bern. Sieg. Albini. Lugd. Batav., 1725. 2 vols. fol.

who by their labors shed an imperishable lustre on the closing years of the sixteenth century. The result was particularly apparent in Italy—in the very spot that had so long echoed with the sounds of applause he had received from his admiring pupils. There indeed anatomy had become an object of worship, and there was formed that admirable school of which he was the chief; and the almost entire number of whose members attained the highest renown—Fallopius, Colombus, Varolius, Aranzi Canani, Vander Spiegel. Vesalius may be said, with apparent justice, to have been not only the creator of normal anatomy, but also to have given a strong impulse to pathological anatomy. In his splendid work on anatomy may be found a large number of pathological facts, which it was his intention to have collected in a separate treatise. The task was reserved for Colombus, who inserted the most important of these facts in his volume *De re anatomica.*

To the same distinguished individual physiology, which received an impulse in the seventeenth century similar to that which anatomy had experienced in the sixteenth, is greatly indebted. Two memorable discoveries date from that period—those of the circulation of the blood and of the lymphatic vessels. M. Burggraeve remarks that if Vesalius remained a stranger to the latter, he may be said to have paved the way to the former by preparing all the elements of the important problem which it was reserved for Harvey to solve.

"Thus if it be true that the knowledge of the circulation of the blood is founded entirely on that of the organs which effect it, who more justly than Vesalius can aspire to the honor of having pointed out those circumstances? He it was who by determining in a precise manner the disposition of the heart, and by describing the admirable mechanism of its valves, indicated to the English physiologist the course he was to pursue in order to affix his name to one of the most important discoveries of modern times. The question was ripe, and Harvey is simply entitled to credit for having seized the *apropos*, inasmuch as it was only necessary for him to draw the natural conclusions flowing from the facts established by the Belgian anatomist.

"Among the authors who illustrated the seventeenth century, Malpighi, Bellini, Lower, Glisson, Santorini, Senac, Duverney, Ruysch, De Graeve, and many others it were too long to enumerate, stand pre-eminent. But even here it will not be difficult to show that Vesalius may be regarded as having guided them in their researches. If all these anatomists became specialists, it is because, finding the field of the science already entirely explored, they were compelled to limit themselves to the cultivation of a small portion of it, in order to be able to make new acquisitions. And even these few cannot be referred to them without some reserve. Thus the germs of the researches of Malpighi on the brain, the liver, and the spleen ; of Willis on neurology; of Senac, Lower, and Winslow on the heart, are to be found in the anatomy of Vesalius."

M. Burggraeve thinks, further, that Vesalius anticipated the views which two hundred years after his time inspired Bordeu and Bichat with the idea of the general anatomy.

The same author is of opinion that in regard to the investigations on comparative anatomy which have assumed so great an extension in the nineteenth century, Vesalius is entitled to a large share of the credit accruing therefrom. It is true, he dispossessed the anatomy of the lower animals of the part which Galen had made it play in the science of the anatomy of man. But, compelled as he was to combat and to exhibit at each step the inaccuracies of that great man, Vesalius was under the necessity of constantly comparing the human body with that of the lower animals, in order to be able to point out the differences existing between them. In other words, in demonstrating in what respect organizations differ from, and in what they resemble, each other, he indicated to the Vicq D'Azyrs, the Daubentons, the Cuviers, the Dumerils, the Blainvilles, the Geoffroy St. Hilaires, the Meckels, the Tiedemanns, the Mullers, the Caruses; in a word, to all those anatomists who in our days trace the analogies of living beings amid the infinite variations they present, the route they were to follow.

On this point the work of Vesalius is admirable. M. Burggraeve tells us that he read it through, Cuvier and Meckel in hand, and did not find him in a single instance guilty of an erroneous statement, or of a faulty application.

We thus perceive that Vesalius may, to a certain extent, be said to have gone through the entire circle of anatomical researches. But it is evident, from all that precedes, that his claims to the undying remembrance of posterity rest almost exclusively on the result of his achievements in the science of normal human anatomy, and on the fact that he was the first to overthrow the ancient prejudices and the blind confidence reposed on the authority of Galen, by exposing boldly and satisfactorily his many anatomical errors. It will not comport with the limits of this essay to enter into anything like a critical review of all the services Vesalius has rendered the science of anatomy. Sufficient is it to remark, that in the department in question his large work constitutes a monument whose fame is not destined to perish for many a long day. It is unlike most other works of science which, as a general rule, have only an importance relative to the period at which they appear. Such, we are reminded by M. Burggraeve, is the march of the human intellect. In proportion as facts are discovered, ideas are modified, and a work which may have been considered of value and complete at the time it was published, ceases, when judged in accordance with the condition of our knowledge on the subject it treats of, to be held otherwise than as a kind of historical document, useful for indicating the difference which exists between the science of that day and the science of our own. This truth is, perhaps, more applicable to works of anatomy than to those relating to any other branch of human knowledge, inasmuch as none have made greater progress or experienced such complete revolutions. In our days no one consults the works of Mundinus, of Zerli, or of Achilini, unless it be as objects of history, and as curious monuments of the infancy of the science. At the side of such works there are others, few it is true, which resist the uninterrupted progressive march of centuries, and preserve eternally an air of freshness and youth—so greatly do they appear to be, as far as regards ideas and facts, contemporaneous with the period at which they are consulted. Such is, and will always be the case with the great work of Vesalius. The long period of three centuries which has passed by since it first appeared has deprived it of none of its actuality, and even at this day it may be considered as the most important monument raised to the science of the organization of man.

Vesalius described in it almost all parts of the body, and only transmitted to his successors the task of correcting a few inaccuracies left in the text and plates from sheer oversight, and not out of regard for Galen; and of adding to it, now and then, a few details which are required to complete the ensemble of the system. To expect entire completeness and perfection in a work of the kind, composed under circumstances such as those that existed at the time, would be folly. Impressed with the belief that the study of an organ cannot be separated from that of its functions, Vesalius was the first to make a satisfactory combination of anatomy and physiology. Speaking of his work he says:—

"I have endeavored to make it a complete treatise of the organization of the human body, and have divided it into seven books.

"In the *first* I describe the nature of the bones and cartilages, as being the parts which anatomists should know first, since it is on them that all the other parts that remain to be known rest and move.

"In the *second* I treat of the parts by means of which the bones and cartilages are joined together; and afterwards of the muscles, organs, of the movements they execute by command of the will.

"The *third* comprehends the veins which carry the blood to all the organs, and the arteries which are charged with the distribution of the vital spirits.

"The *fourth* teaches not only the nerves which carry the animal spirits to the muscles, but also the order in which they originate and are distributed to the organs.

"The *fifth* explains the structure of the organs of nutrition, and, owing to their mutual relations, those of the reproduction of the human race, such as the Creator of all things has established them.

"The *sixth* is appropriated to the heart, source of the vital spirits, and also to the different parts which enter into the structure of that organ.

"Finally, in the *seventh* I have treated of the general harmony of the brain and of the senses, in order that what I have said of the nerves arising from the brain may not be repeated."

As may be perceived, this arrangement does not differ essentially from that adopted in most of the modern works on anatomy. It is similar to those of Bichat, Cloquet, Cruveilhier, and others. According to M. Burggraeve, it is the only natural one, since it enables the writer to describe the parts of the body in accordance with the functions they execute. Nevertheless, Vesalius was fully aware of the necessity of adding to the physiologico-anatomical survey of the body a demonstration of the relations existing between the parts described, and of the order in which the latter present themselves under the scalpel of the anatomist. With the view to attain this object, he devotes, after describing each organ, a chapter to a careful enumeration of those different circumstances. The study of each system of organs is preceded by that of the tissues which enter into their composition; in other words, by what we now denominate their *general anatomy*. Nay more; not content with studying the parts in their normal or physiological conditions, Vesalius examines them as closely as possible in their morbid state, and endeavors to illustrate the facts of normal anatomy by those of pathological anatomy.

Vesalius may be viewed with justice as the creator of anatomical Iconography; for though essays had been made in that department before his time, with the view to supply, by means of drawings, the want of dead bodies, those that had appeared were for the most part coarse, and partook of the infancy of the art. The plates which Vesalius caused to be engraved were like his book itself. They were at least a whole century in advance of their time; for, as M. Burggraeve states, we must reach Ruysch and Albinus before we can find drawings executed with so much vigor and fidelity.

"Those especially which relate to osteology and myology are real masterpieces. They were copied during more than a century by every author who published works on anatomy; and even now, it may be averred that art has produced nothing more perfect."

Of his talent as a practical surgeon or physician, as also of his private character and social virtues, we know but little. That he possessed no great skill as an operator we have already seen there is reason to believe. On the other hand, his work on surgery would seem to attest his proficiency in the other branches of that department. We find in De Thou the account of a case which, if true, shows conclusively that in matters of prognosis he was no child. We give it in De Thou's words.

"Having apprised Maximilian Egmont, Count of Bure, of the day and hour of his death, this nobleman ordered a superb feast to be prepared, and the tables to be covered with all his silver and golden ware. He invited his friends; sat near them; pressed them to make good cheer; distributed liberally among them his treasures. This done, he took leave of them without the least manifestation of emotion; retired to his bed, and expired at the very time Vesalius had predicted."*

Vesalius was married, on his return to Brussels from Italy, to a lady of the name of Clara Van Hanom. But the course of their matrimonial life does not appear to have flowed smoothly—whether through his fault or hers is not positively known—though if the statement of Swartius can be relied upon, the blame must rest upon the lady; for that author attributes the departure of Vesalius from Spain to a desire of escaping from the vexations continually experienced from her ill temper. They left that country together, but parted shortly after; and while he pursued his journey to the Holy Land she proceeded to Brussels, where, shortly after his death, she remarried. Of that marriage the only issue was a daughter, Ann, who married John Moll, the Grand Falconer of the King of Spain.

Delécluse, to whom reference has already been made, lets us into the secret of one of the defects in the character of Vesalius. He represents him, on the authority of Tisenau, as being extremely avaricious.

"But it was his avariciousness that was the cause of his death, as I learned at Madrid, in the month of April, 1565, on my return from Portugal, Andalusia, and the kingdoms of Granada and Valencia. After having fulfilled his vow, Vesalius had embarked to cross again to Europe, which he thought he would soon reach. He did not provide himself with a sufficient quantity of provisions, and, as the passage turned out to be longer than had been expected, they failed him. But one of his fellow-passengers, a German gentleman, having discovered the circumstance, supplied him liberally with means of subsistence. His strength, however, had already been so greatly reduced that he died shortly after reaching the Island of Zanthe."†

When we consider that all that was effected by Vesalius occurred three full centuries ago; that nothing equal to it in any respect had been before seen; that Vesalius succeeded in producing, at a period of life when, with few rare exceptions, men are occupied in acquiring knowledge, a complete work upon the entire anatomy of the human body, derived from actual observations and dissections; when, let it be remembered, such investigations were held to be unlawful; that while scattering to the winds in the face of the most violent opposition an anatomical system which had reigned supreme during some fourteen hundred years, he erected one which, with some modifications that time alone did not permit him to effect, remains

* Historia, etc. vol. i. lib. iv., p. 196. London, 1733.

† De Thou, iii. pp. 491, 492. Fr. trans.

untouched to this day, we may be pardoned for regarding him as occupying one of the highest positions in the medical hierarchy, and as being fully entitled to the time and space we have devoted to an account of the principal events of his life and of his scientific achievements, as also to a vindication of his professional character from imputations of a serious kind.

Portal can scarcely be taxed with an unpardonable degree of exaggeration when, lost in admiration of the genius of our author, he exclaims:—

"Vesale me paroît un des plus grands hommes qui ait existé. Que les Astronomes me vantent Copernic; les Physiciens, Galilée, Toricelli, etc.; les Mathématiciens, Pascal; les Géographes, Christophe Colomb. Je metterai toujours Vésale au-dessus de leurs héros. La première étude pour l'homme, c'est l'homme. Vésale a eu ce noble objet et l'a rempli dignement; il a fait sur lui même et dans les corps de tous ses semblables, des déconvertes que Colomb n'a pu faire qu'en se transportant a l'extrémité de l'univers. Les déconvertes de Vésale touchent directement l'homme; en acquerant de nouvelles connoissances sur sa structure, l'homme agrandit, pour ainsi dire, son existence, au lieu que les déconvertes de Géographie, d'Astronomie ne touchent l'homme que d'une manière très indirecte. La maison de Vésale sert aujourd'hui de Convent aux Capucins de Bruxelles. Ces Religieux se font encore un honneur de dater leurs lettres *ex ædibus Vésalianis.* Ou trouve des gens de goût dans tous les état."*

